# Plant RNA interference from antiviral silencing to multiplex trait engineering for climate-resilient crops

**DOI:** 10.3389/fpls.2026.1871239

**Published:** 2026-07-14

**Authors:** Ibrokhim Y. Abdurakhmonov

**Affiliations:** 1 Center of Genomics and Bioinformatics, Academy of Sciences of Uzbekistan, Tashkent, Uzbekistan; 2 Ministry of Agriculture of Uzbekistan, Tashkent, Uzbekistan

**Keywords:** alternative splicing, biosafety, climate resilience, crop improvement, epigenetics, genome editing, host-induced gene silencing, pangenomics

## Abstract

RNA interference (RNAi) in plants has evolved from an unexplained antiviral and transgene interference phenomenon into a general regulatory platform for sequence-guided gene suppression, chromatin control, systemic signaling, and phenotypic plasticity. This Review synthesizes six decades of plant RNAi, tracing its progression through conceptual bottlenecks and technological solutions. Early work established that RNA-derived homology could suppress viral infection and transgene expression. Mechanistic studies then revealed a diversified plant silencing system involving Dicer-like proteins, Argonautes, RNA-dependent RNA polymerases, systemic movement, and RNA-directed DNA methylation. In parallel, RNAi moved into crop design, enabling targeted modification of yield, fiber quality, flowering, disease resistance, allergenicity, fertility, plant architecture, lignin content, nutrient composition, and pest resistance across diverse species. Importantly, RNAi is not merely a historical precursor to genome editing. It retains distinct value because it can tune gene dosage, silence multigene families, uncover compensatory network responses, and perturb upstream regulatory nodes, such as phytochrome RNAi in cotton, where partial suppression simultaneously improves several negatively correlated traits. Most recently, host-induced silencing, spray-induced dsRNA, nanocarrier delivery, and CRISPR-associated RNA tools have repositioned RNAi as a versatile breeding platform. The future lies in convergence with genome editing, using pangenome-informed, allele-aware target design and combined RNAi-editing pipelines. The lesson learned is that useful crop engineering often requires rebalancing endogenous networks rather than permanent gene knockout. In this review, the historical developmental phases are used carefully: the formal molecular term RNA interference emerged in the late 1990s, while earlier plant work on antiviral resistance, co-suppression and post-transcriptional gene silencing anticipated the same sequence-guided logic. At the same time, practical deployment remains constrained by variable knockdown, off-target risk, construct instability, environmental degradation of sprayed RNA, delivery cost, resistance evolution in target pests or pathogens, regulatory classification, and public acceptance; these constraints are discussed as platform-specific design and risk-assessment issues rather than as generic barriers.

## 1 Introduction

### 1.1 Crop-improvement context

Crop improvement is increasingly judged not only by yield but also by resilience, input efficiency, product quality, and the capacity to perform under climatic instability. In this setting, plant RNA interference (RNAi) is best understood not simply as a gene-silencing technique, but as a sequence-guided regulatory platform that has repeatedly changed the way plant biologists think about causality, plasticity, and trait design (Register and Beachy, 1988; Rao and Hall, 1991; Agrawal et al., 2003; Kusaba, 2004; Baulcombe, 2004; Tenllado et al., 2004). The first phase of the field was descriptive: unusual forms of resistance and homology-dependent suppression were observed before the mechanism was known (Register and Beachy, 1988; Rao and Hall, 1991). The second phase was mechanistic: plants were shown to possess a complex endogenous silencing apparatus involving distinct small-RNA classes, diversified Dicer-like and Argonaute functions, RNA-dependent RNA polymerases, systemic signaling, and chromatin-level silencing (Dalmay et al., 2000; Agrawal et al., 2003; Kusaba, 2004; Baulcombe, 2004; Tenllado et al., 2004; Herr and Baulcombe, 2004; Mathieu and Bender, 2004; Matzke et al., 2009). The third phase was translational: RNAi became a practical route to crop engineering, first for antiviral defense, then for endogenous gene function, and later for metabolic redesign, pest resistance, quality improvement, and developmental rewiring (Lawrence and Pikaard, 2003; Baulcombe, 2004; Tenllado et al., 2004; Kalantidis et al., 2008; Xu et al., 2011; Patil et al., 2011; Lilley et al., 2012; Cillo and Palukaitis, 2014). A fourth phase is now emerging in which RNAi is integrated with exogenous RNA delivery, nanotechnology, CRISPR-derived RNA tools, and pangenome-informed target selection (Ali et al., 2018; Dalakouras et al., 2020; Termolino, 2021; Bao et al., 2021; Kuo and Falk, 2022; Koeppe et al., 2023; Krzyszton et al., 2025).

**Figure 1:**
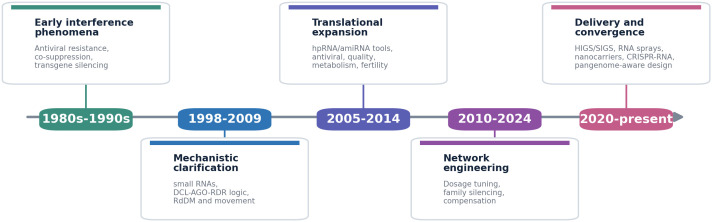
Phase changes in plant RNA interference from early interference phenomena to delivery-enabled RNA-guided platforms. The figure distinguishes early antiviral and transgene-related observations from the formal mechanistic era of RNAi, subsequent engineered crop RNAi, network-level trait design, and modern delivery and convergence approaches. Milestone references are compiled in **Supplementary Table S1**.

This Review develops a “story” of plant RNAi across six decades of plant biology, emphasizing how each phase addressed a distinct bottleneck (**Figure 1**). The central argument is that RNAi has retained value because a combination of pleiotropic effects, dosage sensitivity, and compensatory responses can reveal and rebalance complex regulatory networks. In plants, traits of greatest breeding interest are rarely determined by a single genetic locus acting in isolation. They emerge from connected developmental and stress-response networks. RNAi, especially when applied to regulatory nodes or gene families, can therefore produce coordinated trait rebalancing that is difficult to achieve through conventional breeding and not always desirable with complete gene knockout alone (Xu et al., 2011; Patil et al., 2011; Lilley et al., 2012; Abdurakhmonov et al., 2014).

### 1.2 Scope and historical framing

This review, therefore, fills a practical gap between mechanism-centered RNAi reviews and application-centered crop biotechnology reviews. It asks when RNAi remains scientifically and strategically useful in the era of CRISPR-based genome editing, while distinguishing established field or commercial examples from forward-looking opportunities. Literature analysis refers to a longer plant-biotechnology development that begins with antiviral resistance, transgene-associated silencing and co-suppression, proceeds through PTGS, small-RNA discovery, RdDM and TGS, and culminates in modern hpRNA, amiRNA, VIGS, HIGS, SIGS and nanocarrier-enabled RNA platforms.

## 2 Historical developments: from interference phenomena to defined RNAi

### 2.1 Early antiviral interference and co-suppression

Long before RNAi became a molecular pathway, plant systems had already revealed its logic: antiviral interference and the homology principle. Pathogen-derived resistance experiments showed that transgene-mediated resistance could arise through interference with early events of virus infection, not only through direct protein action (Register and Beachy, 1988). Related work on viral RNAs and replicative interference further suggested that sequence homology and RNA structure can determine infection and replication suppression (Rao and Hall, 1991). These observations, while mechanistically incomplete, established a critical principle: RNA could function as an information-bearing antagonist rather than merely a passive intermediate.

Co-suppression in petunia then showed that introduced transgenes could silence homologous endogenous genes, further demonstrating that homology-dependent gene suppression was a general plant phenomenon rather than a peculiarity of virus resistance (Napoli et al., 1990). The formal discovery that double-stranded RNA triggers potent and specific gene interference in *Caenorhabditis elegans* provided the term and mechanistic grammar of RNA interference (Fire et al., 1998), while the identification of small antisense RNAs in plant PTGS helped connect co-suppression, antiviral silencing, and transgene-mediated suppression within a plant small-RNA framework (Hamilton and Baulcombe, 1999). These early-phase developments have been extensively reviewed elsewhere, including in syntheses of plant RNA silencing, antiviral defense, and RNAi applications in crop functional genomics (Baulcombe, 2004; Cillo and Palukaitis, 2014; Abdurakhmonov, 2016).

### 2.2 Small RNAs, RdDM and pathway definition

Once RNAi was formally recognized as a general eukaryotic mechanism, plant research rapidly became central to its interpretation. Early syntheses made clear that the plant field was not simply borrowing a concept from other organisms; instead, plants revealed an unusually elaborate diversification of RNA silencing into antiviral, developmental, and chromatin-related pathways (Agrawal et al., 2003; Kusaba, 2004; Baulcombe, 2004; Tenllado et al., 2004). RNAi in crop plants was immediately attractive because it promised something that conventional transgenics and classical mutagenesis often struggled to deliver: reversible or partial suppression of endogenous genes, targeted reduction of multigene family activity, and the possibility of engineering resistance or quality traits using sequence information rather than foreign protein-coding functions alone (Agrawal et al., 2003; Kusaba, 2004; Tenllado et al., 2004).

A decisive insight of this period was that plant viral systems were not peripheral examples, but formative experimental models. Reviews on antiviral silencing and viral suppressors demonstrated that virus-host conflict exposed the architecture of the endogenous pathway itself (Baulcombe, 2004; Herr and Baulcombe, 2004; Mathieu and Bender, 2004). Viral suppressor proteins revealed where silencing could be blocked; viral small RNAs revealed how recognition and amplification occurred; and the mobility of silencing across tissues suggested that plant RNAi was linked to long-distance communication in ways that would later become central to crop protection strategies (Dalmay et al., 2000; Herr and Baulcombe, 2004; Mathieu and Bender, 2004; Matzke et al., 2009). In retrospect, the historical importance of these early efforts lies in the fact that plant RNAi emerged from real biological conflicts-virus infection, transgene instability, transposon control-before it became a designed technology. These early phase transitions and the bottlenecks they resolved are summarized in **Table 1**. Selected milestone references that anchor this narrative arc are compiled in **Supplementary Table 1**.

**Table 1:** Plant RNAi as phase-defining problem-solution transitions.

Era/approximate period	Main bottleneck	What RNAi or RNA-silencing research clarified	How the field changed
Early interference phenomena (1980s-1990s)	Virus resistance, transgene effects and co-suppression were observed before a unified mechanism existed.	Sequence homology and RNA-related triggers could interfere with viral infection or homologous gene expression.	Antiviral resistance and gene suppression became interpretable as information-guided processes rather than odd transgene artifacts.
Mechanistic clarification (1998-2009)	PTGS, viral silencing, miRNAs, siRNAs and chromatin silencing appeared heterogeneous.	Small RNAs, DCL/AGO/RDR functions, amplification, systemic spread, and RdDM mechanistically link these phenomena.	RNAi became a framework for development, antiviral defense, genome defense and epigenetic regulation.
Translational expansion (2005-2014)	Model-system success needed to be converted into crops.	hpRNA, amiRNA and crop transformation pipelines enabled antiviral, metabolic, developmental and quality engineering.	RNAi moved from Arabidopsis and tobacco into cassava, rice, soybean, rapeseed, cotton, grasses and horticultural crops.
Network engineering (2010-2024)	Single-gene logic was insufficient for multigenic traits.	Partial suppression of regulators, gene families and pathway nodes revealed dosage responses and compensation.	The field learned that partial suppression can reveal and exploit network rebalancing rather than the simple loss of function.
Delivery and convergence (2020-present)	Stable transgenics alone were too narrow for some agricultural uses.	HIGS, SIGS, nanocarriers, RNA biopesticides and CRISPR-linked silencing broadened deployment options.	RNAi became part of a wider RNA-guided innovation platform for crop protection and breeding.

The discovery of endogenous and silencing-associated small RNAs in plants, and the identification of plant microRNAs, further clarified that RNAi encompasses both siRNA-mediated silencing and miRNA-mediated endogenous regulation (Llave et al., 2002; Reinhart et al., 2002). In parallel, promoter dsRNA-triggered transcriptional silencing and methylation showed that plant RNAi-like processes also operate at the chromatin level through RdDM and TGS (Mette et al., 2000; Mathieu and Bender, 2004; Matzke et al., 2009).

## 3 RNAi became a mechanistic language for plant biology

### 3.1 DCL, AGO and RDR specialization

The major conceptual shift of the 2000s was the realization that plant RNAi was not one pathway but a modular regulatory system. Core mechanistic studies defined the roles of Dicer-like proteins, Argonautes, RNA-dependent RNA polymerases, and plant-specific transcriptional machinery that together supported post-transcriptional silencing, systemic spread, and RNA-directed DNA methylation (Dalmay et al., 2000; Tenllado et al., 2004; Herr and Baulcombe, 2004; Mathieu and Bender, 2004; Matzke et al., 2009; Agius et al., 2012). The discovery that an RNA-dependent RNA polymerase is required for transgene-mediated post-transcriptional silencing in Arabidopsis clarified that plants possess amplification capacity, allowing silencing to spread beyond the initial trigger (Dalmay et al., 2000). At the same time, work on RNA-directed DNA methylation and plant-specific polymerase functions has shown that RNAi in plants extends to chromatin, transposon control, and transcriptional memory (Mathieu and Bender, 2004; Matzke et al., 2009; Agius et al., 2012; Dalakouras et al., 2009).

In practical terms, DCL1 mainly processes miRNA precursors, DCL2 and DCL4 contribute strongly to antiviral and post-transcriptional siRNA pathways, and DCL3 produces 24-nt siRNAs associated with RdDM. AGO1 is central to miRNA and many PTGS reactions, AGO2 contributes to antiviral defense and stress responses, and AGO4 is strongly associated with 24-nt siRNAs and chromatin-level silencing. RDR1 and RDR6 participate in antiviral and secondary siRNA pathways, whereas RDR2 functions with plant-specific RNA polymerase IV in RdDM. This specialization helps explain why 21-nt, 22-nt and 24-nt small RNAs often differ in mobility, amplification and chromatin effects.

### 3.2 Small-RNA pathway routing and systemic movement

This mechanistic diversification made plants uniquely informative. In addition to canonical microRNAs and small interfering RNAs, the plant field revealed secondary siRNA production, transitivity, systemic silencing, and multiple tiers of movement between cells and organs (Herr and Baulcombe, 2004; Kalantidis et al., 2008; Mermigka et al., 2016). These discoveries changed the meaning of “silencing efficiency.” The strongest RNAi response was not always the one with the greatest local knockdown, but often the one with the most effective entry into amplification, movement, or transcriptional reinforcement. That insight later influenced the design of hairpin constructs, artificial microRNAs (amiRNAs), and delivery systems for both endogenous genes and invading pathogens (Lawrence and Pikaard, 2003; Kerschen et al., 2004).

Mechanistic work also exposed an intimate relationship between RNAi and RNA quality control. Defects in processing, maturation, or decay can channel transcripts into silencing pathways, and recent syntheses continue to emphasize that mRNA turnover and small-RNA biogenesis are tightly balanced processes rather than separate regulatory universes (Krzyszton et al., 2025). This theme is historically important because it moved the field beyond a simple “destroy the target mRNA” model. RNAi began to be understood as part of a broader cellular logic that distinguishes productive transcripts from aberrant, invasive, or excessive RNA molecules. In plants, where environmental stress continuously reshapes transcription, this coupling between turnover and silencing provided a conceptual bridge between development, defense, and adaptation.

## 4 From silencing to regulation: endogenous networks, epigenetics, and RNA processing

As the field matured, RNAi ceased to be viewed only as a defense mechanism and increasingly became a framework for endogenous regulation. RNA-mediated chromatin silencing in plants showed that small RNAs guide methylation and higher-order epigenetic states, helping to stabilize genome integrity while also contributing to development, transposon control, and phenotypic variation (Mathieu and Bender, 2004; Matzke et al., 2009; Dalakouras et al., 2009). This broadened the significance of RNAi in two ways. First, it embedded RNAi within the architecture of plant genomes rather than treating it solely as a response to external factors. Second, it suggested that RNAi can produce phenotypic consequences that extend beyond a single target gene or transcript class, because chromatin and transcriptional reprogramming can affect broader regulatory territories.

A related advance was the appreciation that RNAi interfaces with non-coding RNAs, precursor structure, and RNA processing steps that influence which strands are loaded, amplified, moved, or converted into secondary siRNAs (Kerschen et al., 2004; Iwakawa et al., 2021; Termolino, 2021; Kuo and Falk, 2022). Guide-strand choice, precursor architecture, and secondary siRNA production are not trivial biochemical details; they are determinants of biological output. Studies on amiRNA design, precursor features, and ribosome-linked secondary siRNA production all reinforced the idea that RNAi can be engineered not only at the level of sequence complementarity, but also at the level of pathway routing (Termolino, 2021; Kuo and Falk, 2022). This engineering logic is especially relevant for crop applications, because a silencing trigger can be designed to favor specificity, breadth, mobility, or durability depending on the target problem.

Another frontier concerns alternative splicing and other transcript-processing decisions. The plant literature increasingly points to reciprocal interactions between RNA silencing, immune regulation, and alternative splicing, especially under biotic stress (Zhu et al., 2025). Here, the conceptual value for breeding is substantial. If stress adaptation is mediated partly by isoform choice, RNA turnover, and small-RNA pathway activity, then RNAi is not merely a tool to knock down a gene; it becomes a way to probe and potentially redirect regulatory plasticity. This matters in the context of climate change, where rapid adjustments in developmental timing, stress signaling, and defense allocation may depend on dosage-sensitive regulatory circuits rather than simple on/off mutations. In this sense, pleiotropy is not an inconvenience to be eliminated. It often serves as evidence that the intervention has engaged a genuine control node.

## 5 RNAi moved from model systems to crop design

### 5.1 Protection, quality and metabolism

The translational expansion of plant RNAi was remarkably fast. Antiviral engineering provided the first major proof-of-principle, with hairpin RNAs and, later, amiRNAs used to confer resistance in diverse crops (Baulcombe, 2004; Patil et al., 2011; Yadav et al., 2011; Jelly et al., 2012; Cillo and Palukaitis, 2014). Cassava brown streak disease is a strong example of the field’s practical reach: RNAi constructs targeting viral coat-protein sequences produced robust resistance in both model and crop contexts, confirming that small-RNA accumulation can be translated into durable antiviral protection in a major food-security crop (Yadav et al., 2011; Jelly et al., 2012). Similar logic extended to resistance against insects and nematodes, where plant-mediated silencing of pest genes conferred resistance phenotypes that conventional host resistance did not readily confer (Lilley et al., 2012; Xiong et al., 2019; Saakre et al., 2024).

At the same time, RNAi expanded from protection to quality and metabolism. Silencing caffeine biosynthesis opened the route to decaffeinated coffee (Ogita et al., 2003; Glenn et al., 2013); seed-specific RNAi of the gossypol pathway generated ultra-low-gossypol cottonseed while preserving the capacity to resume terpenoid profiles after germination and showing generational stability of the RNAi-mediated phenotype (Sunilkumar et al., 2006; Rathore et al., 2012); silencing allergen genes reduced allergenicity in carrot (Peters et al., 2011); silencing storage-protein genes altered rice grain composition (Cho et al., 2016); and suppression of fatty acid desaturase genes generated high-oleic soybean (Wang and Xu, 2008).

### 5.2 Developmental and multi-trait engineering

Developmental engineering was equally revealing. RNAi knockdowns of flowering, meristem, hormonal, and fertility regulators in rice, strawberry, rapeseed, and Arabidopsis repeatedly showed that partial gene suppression can reshape architecture, fertility, or developmental timing without reproducing the lethality or rigidity expected from null mutations (Schwab et al., 2006; Cui et al., 2010; Chatterjee et al., 2011; Abdurakhmonov et al., 2014; Liu and Ding, 2024; Li et al., 2025). This is where RNAi’s breeder-facing logic becomes especially strong. Many of the most useful agronomic targets sit high in developmental hierarchies. Complete loss of function may be catastrophic, but partial, tissue-biased, or family-level suppression can rebalance growth. The most striking illustration from this perspective is phytochrome RNAi in cotton, developed by our group. In that system, suppression of *PHYA1* by up to approximately 70% triggered compensatory overexpression of other phytochromes, while improving fiber length and related fiber traits, advancing flowering and maturity, strengthening root and vegetative growth, and increasing seed cotton yield on average by 10–17% relative to controls (Abdurakhmonov et al., 2014, 2016; Miao et al., 2017; Kamburova et al., 2022; Abdurakhmonov et al., 2026a, 2026b). Importantly, this case also raises a broader translational issue: whether RNAi-derived phenotypes remain stable over the long term during agricultural deployment (Abdurakhmonov et al., 2026a, preprint). In plants, RNAi has become a powerful tool for trait engineering. Still, the durability of beneficial RNAi phenotypes under multi-season field conditions remains less well documented than short-term proof-of-concept efficacy. This question is especially relevant for transgene-based hairpin RNA systems because conventional hpRNA constructs are prone to intrinsic DNA methylation and transcriptional self-silencing, thereby reducing the stability of RNAi output over time. Recent work has shown that introducing nucleotide mismatches into hpRNA constructs can prevent intrinsic self-silencing and enhance RNAi stability, underscoring that the trigger architecture itself is an important determinant of phenotypic durability (Zhang et al., 2022). Against this background, as reported in a recent preprint, our 13-year field observations suggest that, in elite cotton cultivars under practical production conditions, the associated yield and fiber-quality advantages may remain stable over time (Abdurakhmonov et al., 2026a, preprint). These results do not eliminate the broader risk of hpRNA instability across species, loci, or construct classes. Still, they suggest that well-designed RNAi events may retain agronomic value after commercial deployment. In that sense, the cotton phytochrome case is conceptually important not only because it demonstrates that RNAi can overcome classical negative correlations by perturbing an upstream developmental regulator rather than by optimizing each downstream trait separately, but also because it provides rare long-term field support for the durability of a complex RNAi-derived phenotype.

The network-rebalancing claim is strongest when RNAi studies document more than target transcript reduction. Lignin and cell-wall RNAi show pathway-level effects through altered lignin amount or composition, digestibility and saccharification; flowering and meristem examples show dosage-sensitive developmental outputs; and *PHYA1* RNAi in cotton links partial suppression to compensatory phytochrome expression, altered microRNA profiles and antioxidant system activity (Miao et al., 2017; Kamburova et al., 2022). These examples support a restrained conclusion: RNAi can reveal and sometimes exploit network rebalancing when the target is a genuine regulatory or pathway node and when molecular, physiological and agronomic evidence are considered together.

**Figure 2:**
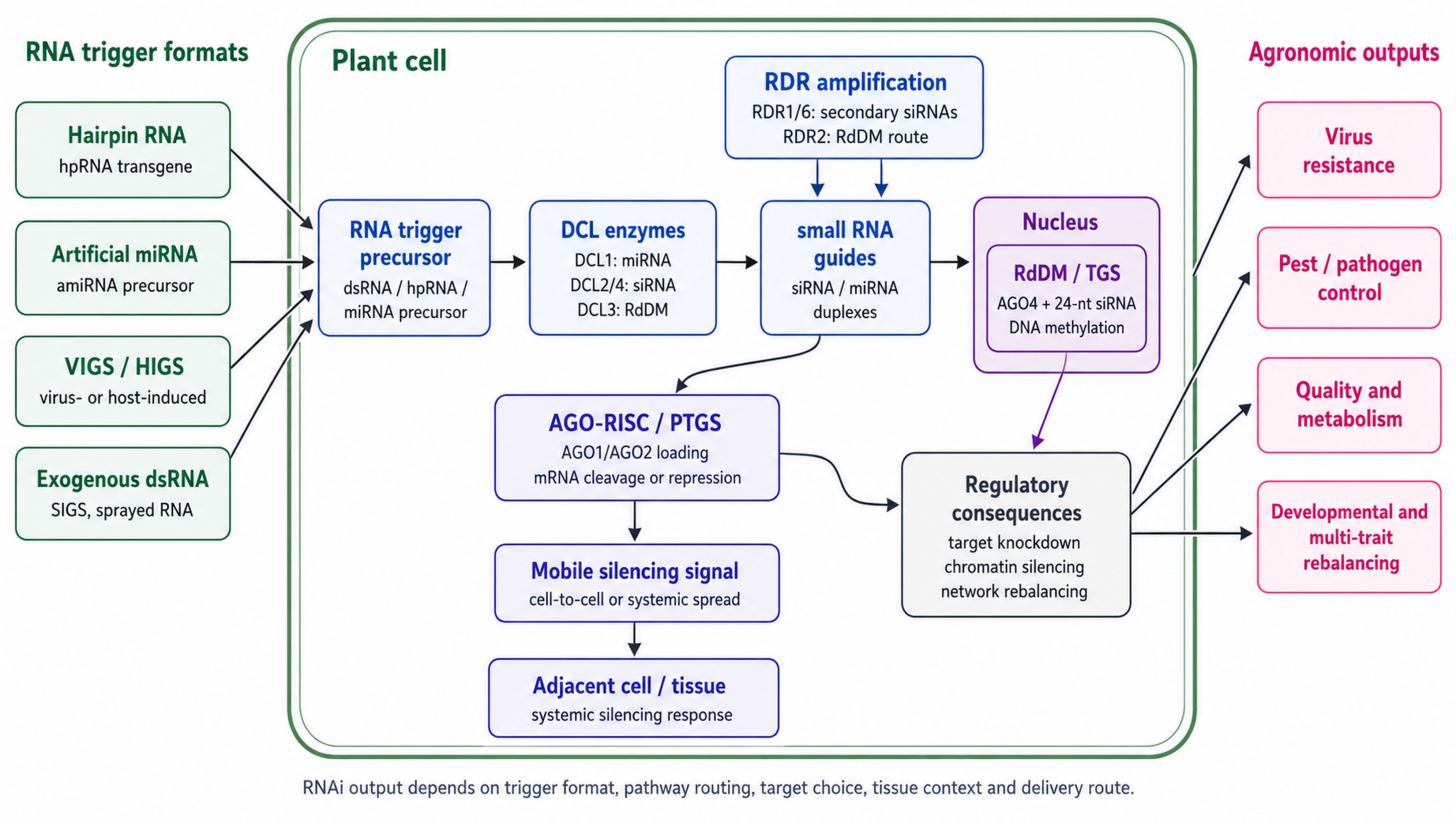
From RNA trigger to agronomic phenotype. Trigger format influences pathway entry, pathway routing, and the final biological output of RNAi. Hairpin RNA (hpRNA), artificial microRNA (amiRNA), virus- or host-induced gene silencing (VIGS/HIGS), and exogenous double-stranded RNA used in spray-induced gene silencing (SIGS) can engage post-transcriptional silencing, RNA-directed DNA methylation, amplification, systemic movement, or broader regulatory responses. The routes shown are simplified, illustrative pathways rather than fixed one-to-one relationships. Depending on target choice, trigger design, tissue context, and delivery route, RNAi outcomes may range from single-gene knockdown to pest or pathogen control, quality modification, stress or developmental reprogramming, and coordinated multi-trait rebalancing.

The mechanistic logic by which trigger architecture is translated into regulatory routing and, ultimately, agronomic phenotype is illustrated in **Figure 2**. Representative crop exemplars that explain why RNAi has remained strategically valuable across protection, metabolism, development, and multi-trait engineering are summarized in **Table 2**.

**Table 2:** Crop exemplars showing evidence level, limitations and key references for RNAi applications.

Crop/system	Target/strategy	Validation and reported effect	Limitations or remaining challenges	Key references
Cassava	Viral coat-protein sequences from cassava brown streak viruses; transgenic hpRNA/pathogen-derived RNAi	Greenhouse and crop-context validation: strong resistance to diverse viral isolates in transgenic cassava lines.	Virus diversity, regulatory approval, farmer access and durability under varied agroecologies remain important.	Patil et al., 2011; Yadav et al., 2011
Coffee	Caffeine biosynthesis pathway; transgenic RNAi of caffeine pathway genes	Plant proof-of-concept: decaffeinated coffee plants generated through metabolic downregulation.	Commercial deployment remains constrained by transformation, acceptance and quality/stability validation.	Ogita et al., 2003; Glenn et al., 2013
Carrot	Dau c 1.01 and Dau c 1.02 allergen genes; transgenic RNAi	Plant and immunological validation: reduced allergenic reactivity in silenced carrot plants.	Consumer acceptance and regulatory pathway for allergen-reduced transgenic food crops remain challenging.	Peters et al., 2011
Rice	Seed storage-protein genes; RNAi-mediated simultaneous suppression	Plant proof-of-concept: simultaneous suppression of modified storage-protein accumulation.	Nutrition, processing quality, yield penalty and multi-environment stability require assessment.	Cho et al., 2016
Soybean	GmFAD2 fatty-acid desaturase family; transgenic RNAi	Transgenic plant validation: increased oleic-acid profile through suppression of desaturase activity.	Event stability, regulatory route and comparison with edited high-oleic alleles should be considered.	Wang and Xu, 2008
Switchgrass, sugarcane, Brachypodium and other grasses	4CL, COMT, HCT, CCoAOMT and SAMS-related wall pathways; RNAi/hpRNA	Greenhouse to field-grown material depending on study: reduced lignin, improved digestibility or saccharification, sometimes with manageable growth penalties.	Growth penalties, lodging, stress response and environment-dependent biomass performance must be monitored.	Xu et al., 2011; Jung et al., 2013; Serrani-Yarce et al., 2021; Li et al., 2022
Rapeseed and reproductive systems	Fertility and metabolic male-sterility pathways; RNAi-based developmental engineering	Plant proof-of-concept: RNAi created controllable fertility-related phenotypes.	Fertility systems require stability, containment and restoration logic for breeding use.	Chatterjee et al., 2011; Abdurakhmonov et al., 2016
Rice, strawberry and Arabidopsis developmental models	Floral homeotic, KNOX, miRNA/amiRNA and developmental regulators	Model and crop proof-of-concept: partial suppression changed meristem, floral or developmental traits.	Translation to breeding depends on avoiding sterility, yield penalty or unstable developmental effects.	Schwab et al., 2006; Cui et al., 2010; Chatterjee et al., 2011
Cotton	PHYA1 light-signaling regulator; transgenic *PHYA1* RNAi	Greenhouse, field trials and long-term field observations/preprint: partial suppression associated with compensatory phytochrome expression, fiber-quality improvement, earlier maturity, vegetative vigor and 10-17% yield advantage.	Broader multi-location validation, independent replication, regulatory status and peer-reviewed long-term evidence remain important.	Abdurakhmonov et al., 2014, 2016; Miao et al., 2017; Kamburova et al., 2022; Abdurakhmonov et al., 2026a
Common bean	Whitefly-related pest target; transgenic/host-delivered RNAi	Transgenic line validation: improved tolerance to whitefly in common bean.	Target specificity, resistance management, regulatory route and non-target assessment remain necessary.	Ferreira et al., 2022
Botrytis and fungal pathosystems	BcTOR, DCL/CYP51 and other fungal genes; HIGS or SIGS with dsRNA/sRNA	Greenhouse, detached tissue and field-relevant evaluations: reduced fungal development or increased resistance when pathogen genes were silenced.	Uptake variability, fungal strain variation, environmental stability, formulation and field persistence limit translation.	Wang et al., 2016; Koch et al., 2016; Xiong et al., 2019; Mann et al., 2023; Capriotti et al., 2024
Maize/western corn rootworm	DvSnf7 and related insect targets; plant-incorporated RNAi trait	Commercial/regulatory deployment: RNAi trait reached commercial crop-protection use against western corn rootworm.	Resistance management, stewardship, non-target assessment and regulatory monitoring remain central.	U.S. EPA, 2017; Darlington et al., 2022
Potato/Colorado potato beetle	PSMB5 in Colorado potato beetle; sprayable dsRNA active ingredient ledprona/Calantha	Regulatory approval and commercialization in the USA: a milestone for sprayable dsRNA pesticide use in potato pest control.	Persistence, application timing, resistance management and cost- and jurisdiction-specific approvals remain important.	Rodrigues et al., 2021; U.S. EPA, 2023a; U.S. Federal Register, 2023; Graser et al., 2025

## 6 Why RNAi can outperform or complement single-gene editing in some breeding contexts

The emergence of CRISPR-based editing has sometimes encouraged a misleading historical interpretation that treats RNAi as an imprecise predecessor destined for replacement. That interpretation is too narrow. In plants, RNAi and editing address overlapping but distinct problems (Ali et al., 2018; Dalakouras et al., 2020; Termolino, 2021; Bao et al., 2021; Koeppe et al., 2023). Genome editing excels when a discrete causal locus is known, and a stable DNA change is desired. RNAi remains especially powerful when the objective is dosage modulation, simultaneous targeting of several related genes, reversible testing of essential loci, or the exploitation of compensatory and pleiotropic network responses (Agrawal et al., 2003; Lawrence and Pikaard, 2003; Abdurakhmonov et al., 2014; Termolino, 2021).

This distinction is already explicit in practical crop work. In tomato, RNAi continues to be used because it enables graded downregulation, facilitates the study of life-essential genes, and can suppress multigene families in ways that cannot always be replicated by a single editing event (Termolino, 2021). The same is true in polyploids and other genetically redundant plant systems, where transgene-induced RNA interference was early recognized as a means of overcoming redundancy (Lawrence and Pikaard, 2003). By changing the dosage rather than permanently deleting the function, RNAi can reveal “response surfaces” rather than a single binary phenotype. For breeding, this is highly relevant: some of the best agronomic outcomes arise not from maximal suppression, but from partial rebalancing. The cotton phytochrome case illustrates this clearly, but analogous logic appears in lignin engineering, flowering-time regulation, and stress-signaling studies, where moderate suppression can decouple trade-offs more effectively than full knockout (Xu et al., 2011; Jung et al., 2013; Abdurakhmonov et al., 2014; Serrani-Yarce et al., 2021; Li et al., 2022).

RNAi also offers a different philosophy of multiplexing. Artificial microRNAs, polycistronic tRNA-amiRNA systems, and hairpin designs can simultaneously target several family members or several pest genes (Bao et al., 2021; Kuo and Falk, 2022). This is not simply a convenience. It enables coordinated movement within a regulatory network, precisely where complex traits reside. Editing can, of course, be multiplexed as well, but RNAi has an advantage when the desired output is coordinated weakening rather than irreversible elimination. For some climate-relevant traits, developmental timing, growth-defense balance, wall composition, nutrient use, or stress-signaling crosstalk, the most useful phenotype may arise from a softer intervention. In that specific sense, RNAi can be an alternative to single-gene editing: it is often an efficient way to tune a network rather than remove a part.

## 7 RNAi enters the era of delivery, commercialization and RNA-guided approaches

### 7.1 HIGS and SIGS

The convergence of delivery and platforms has defined the most recent phase of plant RNAi. Host-induced gene silencing (HIGS) and spray-induced gene silencing (SIGS) extended RNAi beyond stably transformed plants. They established the feasibility of RNA-mediated control of fungi, insects, mites, nematodes, and viruses (Lilley et al., 2012; Dalakouras et al., 2020; Bao et al., 2021; Dietz-Pfeilstetter et al., 2021; Ferreira et al., 2022; Mann et al., 2023; Koeppe et al., 2023; Saakre et al., 2024; Graser et al., 2025; Li et al., 2026). Exogenous dsRNA is attractive because it can be specific and programmable, is more environmentally compatible than many conventional chemistries, and, in some cases, can be used without requiring stable transgene integration (Dalakouras et al., 2020; Bao et al., 2021). The central bottlenecks are now stability, uptake, systemic mobility, persistence, and cost. Recent work on formulation, environmental assessment, efficacy, and commercialization shows how rapidly this engineering layer is progressing (Dietz-Pfeilstetter et al., 2021; Mann et al., 2023; Graser et al., 2025; Li et al., 2026). Importantly, these delivery advances have repositioned RNAi from an exclusively transgenic platform toward a broader agricultural technology landscape that includes topical biopesticides, transient functional genomics, and hybrid molecular-control strategies.

HIGS and SIGS nevertheless solve different deployment problems. HIGS uses the plant to produce silencing triggers and can provide sustained RNA exposure at feeding or infection interfaces, but it is typically implemented as a transgenic approach and must address event stability, expression durability, cross-kingdom movement, and resistance evolution in the target organism. SIGS leaves the crop genome unmodified and is attractive for seasonal or non-transgenic protection, but it is limited by nuclease degradation, poor uptake in some target organisms, UV exposure, rainfall, formulation cost, persistence and field-scale efficacy (Nowara et al., 2010; Koch et al., 2016; Wang et al., 2016; Dietz-Pfeilstetter et al., 2021; Mitter et al., 2017; Niño-Sánchez et al., 2022).

### 7.2 Commercialization and the laboratory-to-field gap

This laboratory-to-field gap should be made explicit. RNAi crops or products must move from proof of silencing to event selection, delivery optimization, environmental fate analysis, non-target assessment, resistance-management planning and regulatory review. Commercial milestones show that this is possible: in planta RNAi has reached commercial deployment for western corn rootworm management in maize, and the U.S. Environmental Protection Agency registered ledprona/Calantha, a sprayable dsRNA product against Colorado potato beetle, in 2023 (U.S. EPA, 2017; Darlington et al., 2022; Rodrigues et al., 2021; U.S. EPA, 2023a; U.S. Federal Register, 2023). These examples demonstrate practical progress, but they should not imply that all HIGS, SIGS or RNA biopesticide applications are already field-ready.

## 8 RNAi, CRISPR and pangenome-informed breeding

### 8.1 Complementarity with genome editing

At the same time, CRISPR-associated RNA tools and CRISPR interference have redrawn the boundaries between “editing” and “silencing” (Ali et al., 2018). RNA-targeting Cas systems, dCas-based transcriptional repression, and programmable silencing suggest that future crop engineering will increasingly combine DNA-level and RNA-level interventions rather than choose between them (Ali et al., 2018). This convergence strengthens, rather than weakens, the rationale for RNAi. Once crop improvement is viewed as a problem of regulatory design, multiple layers of intervention become complementary: genome editing can install stable alleles, while RNAi can test dosage space, silence families, and provide non-transgenic or seasonal control options.

CRISPR interference (CRISPRi) and CRISPR activation (CRISPRa) are conceptually close to RNAi-based strategies because they repress or activate expression without permanently altering the coding sequence. However, CRISPRi and CRISPRa operate at the transcriptional level by targeting DNA through a catalytically dead Cas protein, whereas RNAi acts post-transcriptionally by targeting mRNA. Both approaches are useful for dosage modulation and functional screening. In contrast, base editing and prime editing are strongest when a precise, heritable DNA change is desired, but they remain constrained by delivery, editing efficiency, and genotype dependence in many crops (Rajput et al., 2021; Vats et al., 2024; Gallo et al., 2025). RNAi can therefore serve as a front-end discovery tool to identify optimal target genes and dosage windows before committing to permanent genome editing.

### 8.2 Pangenome-informed target design

**Figure 3:**
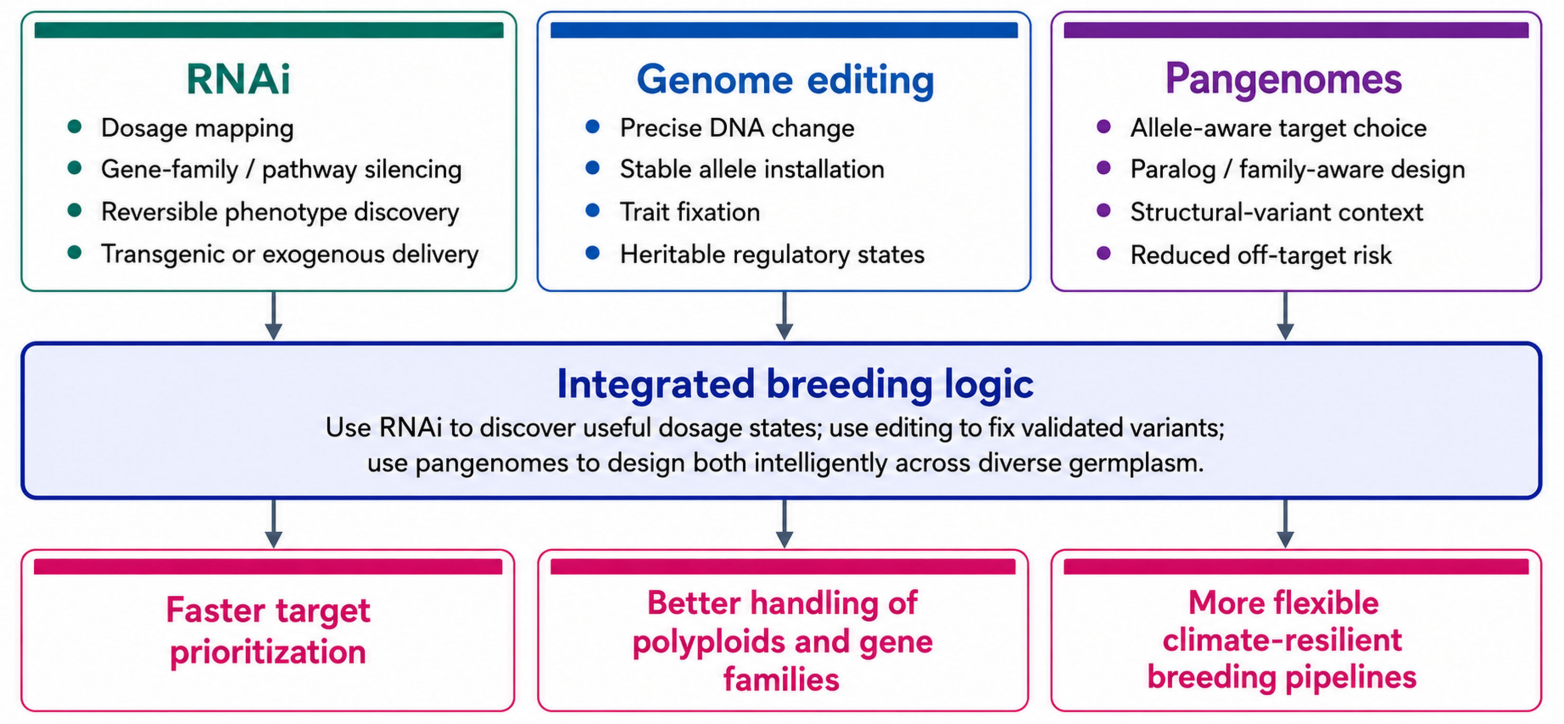
RNAi, genome editing, and pangenome-informed breeding are complementary. RNAi is especially strong for dosage control, multigene suppression, and phenotype discovery; editing is strongest for fixing preferred alleles; pangenomes improve both by providing allele- and family-aware target design. RNAi is best deployed first to map the optimal degree and tissue specificity of suppression across phenotypic dosage space, after which editing or conventional breeding can fix the preferred allelic state in commercial germplasm. Pangenome resources additionally enable allele-aware and family-aware trigger design, reducing off-target risk and improving cross-variety efficacy for both RNAi and editing approaches.

The pangenomics era adds a new dimension. Many crop targets now exist as allelic series, copy-number variants, structural haplotypes, or gene-family expansions across diverse germplasm. This context should favor RNAi if target design becomes allele- and family-aware rather than reference-genome-naive. Pangenomes can help identify conserved trigger windows for broad-spectrum control, while also warning against off-target complementarity in non-target homologs. In parallel, a better understanding of precursor architecture, strand loading, and pathway routing should make amiRNA and dsRNA design more predictable (Iwakawa et al., 2021; Kuo and Falk, 2022). The likely future is therefore not a return to generic “hairpin silencing,” but a more precise RNA-guided engineering framework in which delivery chemistry, sequence design, pathway selection, and genomic diversity are optimized together. The breeding and research situations in which RNAi remains preferable or strongly complementary to single-gene editing are summarized in **Table 3**. This complementarity between dosage-based silencing, allele fixation, and diversity-aware target design is illustrated in **Figure 3**.

**Table 3:** Situations where RNAi may be preferable or complementary to single-gene editing.

Breeding or research situation	Why RNAi is useful	Typical design logic	Editing/pangenome complement
Essential genes or strong developmental regulators	A complete knockout may be lethal or too severe	Use partial knockdown to map useful dosage windows	Use editing only after a favorable dosage or allele is validated
Multigene families or polyploid targets	One edit may not reduce function across homologs	Design one or several triggers for family-level suppression	Use pangenomes to distinguish conserved target windows from off-target paralogs
Network-balanced traits	The desired phenotype may arise from rebalancing, not removal	Silence an upstream regulator and measure compensatory responses	Edit or breed alleles after RNAi identifies the useful network state
Rapid functional screening	Permanent edits are inefficient when the target confidence is low	Use VIGS, amiRNA, transient RNAi, or dsRNA application	Prioritize high-confidence targets for editing or breeding
Seasonal crop protection	Stable transformation may be slow, impractical, or socially constrained	Use SIGS or formulated dsRNA against pest/pathogen genes	Use genomic resources to avoid non-target matches and resistance-prone targets
Tissue-biased or reversible intervention	Permanent edits can be too rigid.	Use promoter choice, trigger architecture, or delivery route to tune silencing.	Use pangenomes and phenomics to refine target context across germplasm.

For both RNAi and editing, pangenomes can reduce design errors by revealing paralogs, presence/absence variation and cultivar-specific sequence polymorphisms that may affect either efficacy or off-target risk (Raza et al., 2023; Schreiber et al., 2024).

## 9 RNAi for climate-resilient crop design

### 9.1 Specific trait framework

Because climate resilience appears in the title, it should be treated as a specific trait framework rather than as a general promise. RNAi can contribute through pathogen and pest resistance under shifting climates, developmental timing, reproductive stability, root and water-use traits, cell-wall remodeling, nutrient-use pathways, and stress-responsive hormone or redox circuits. The evidence is strongest for protection traits and more uneven for abiotic stress tolerance.

### 9.2 Abiotic stress examples and evidence levels

Concrete abiotic examples illustrate the opportunity while also showing why caution is necessary. In rice, RNAi-mediated suppression of OsGRXS17 improved drought tolerance by modulating ROS accumulation and stomatal closure (Hu et al., 2017). Silencing of OsSYT-5 improved rice tolerance to water deficit, accompanied by changes in photosynthesis, stomatal conductance, root architecture, ABA content and the expression of ABA-related stress genes (Shanmugam et al., 2021). RNAi-mediated suppression of OsBBTI5 promoted salt stress tolerance in rice seedlings by modulating ROS-related enzymes, photosynthesis-related genes, and hormone-related signaling pathways (Lin et al., 2024). These examples are valuable proof-of-concept or controlled-condition studies; they should be separated from multi-environment field evidence for yield stability.

For drought, salinity and heat tolerance, nutrient-use efficiency and root architecture, future RNAi studies should report three levels of evidence: molecular evidence that the target regulates stress responses, greenhouse evidence that RNAi improves tolerance and multi-environment field evidence that the phenotype improves yield or quality stability without unacceptable trade-offs. This distinction helps avoid overstating climate-resilience claims while still recognizing the value of RNAi for target discovery.

## 10 Limitations, biosafety and societal considerations

### 10.1 Technical and biological limitations

A balanced view of plant RNAi must begin with its limitations. Knockdown can be incomplete and may vary across tissues, developmental stages, environments, genotypes, construct positions and target transcript abundance. Pleiotropic effects can be useful when a true regulatory node is engaged, but they can also produce undesirable developmental, reproductive, metabolic or stress-response changes. Off-target silencing is a concern when siRNAs match unintended genes, paralogs or non-target organisms. Gene-family redundancy can be either an advantage, when simultaneous suppression is desired, or a challenge, when specificity is needed.

### 10.2 Durability and resistance evolution

Durability is also a first-class design criterion. hpRNA constructs may become progressively weakened by intrinsic DNA methylation or transcriptional self-silencing, although trigger redesign can reduce this risk (Zhang et al., 2022). In cross-kingdom systems, pests and pathogens may evolve reduced uptake, altered target sequences, enhanced nuclease activity or compensatory pathways; this is why resistance management is relevant for both transgenic and externally applied RNAi approaches. For sprayed dsRNA, environmental instability, UV exposure, rainfall, plant-surface retention and biological uptake can determine whether a laboratory result translates to field control.

### 10.3 Regulatory and biosafety considerations

Biosafety and regulation differ by platform. Transgenic RNAi crops are usually assessed as genetically modified plants or plant-incorporated protectants, whereas externally applied dsRNA products are evaluated as pesticides or biopesticides depending on jurisdiction. Risk assessment should consider exposure, degradation, sequence specificity, potential non-target effects, effects on related taxa, environmental fate and the role of formulation in promoting uptake (Romeis et al., 2020; OECD, 2020; Dietz-Pfeilstetter et al., 2021; OECD, 2023). Public acceptance will also differ between stable RNAi crops, seasonal RNA sprays and genome-edited products. Transparent terminology is therefore part of responsible communication, not a cosmetic addition.

## 11 Lessons learned and anticipated developments

Several lessons emerge from the history of plant RNAi. First, the field advanced whenever it treated unusual phenotypes not as noise but as information. Viral recovery, transgene instability, systemic spread, compensation after partial knockdown, and pleiotropic developmental responses all provided mechanistic insights. Second, the most durable applications came from alignment between the mechanism and the target problem. Antiviral RNAi succeeded because plants already deploy RNA silencing against viruses. Lignin redesign succeeded because wall biosynthesis tolerates partial rebalancing better than complete blockage. Developmental RNAi proved most useful where upstream regulators could be tuned rather than erased (Baulcombe, 2004; Mathieu and Bender, 2004; Xu et al., 2011; Yadav et al., 2011; Chatterjee et al., 2011; Jung et al., 2013; Abdurakhmonov et al., 2014; Serrani-Yarce et al., 2021; Li et al., 2022). Third, RNAi repeatedly showed that crop engineering is often most successful when it operates through endogenous regulatory logic. Practical design principles distilled from these historical lessons are summarized in **Supplementary Table 2**. That is why RNAi can “use the plant’s own genes” in a broader and deeper sense than the phrase is sometimes taken to mean: it exploits native small-RNA, amplification, transport, and chromatin machinery to redirect pre-existing biological circuits. A related lesson is that durability must be treated as a first-class design criterion in RNAi breeding, particularly for hpRNA-based transgenes, because construct architecture, epigenetic robustness, and long-term field validation all influence whether an initially successful RNAi phenotype remains agronomically useful (Zhang et al., 2022; Abdurakhmonov et al., 2026a).

Looking ahead, three areas stand out as especially important. One is network-aware target choice. The greatest future gains may come from developmental and signaling hubs whose moderate suppression can deliver several agronomic improvements at once, especially in crops facing combined stress, shortened seasons, and quality demands. A second is isoform- and pathway-aware design, including the integration of RNAi with alternative splicing, transcript decay, and stress-responsive regulatory layers (Krzyszton et al., 2025; Zhu et al., 2025). A third is deployment realism. SIGS, HIGS, and RNA biopesticides will succeed only if stability, formulation, affordability, and ecological validation improve together (Dalakouras et al., 2020; Bao et al., 2021; Dietz-Pfeilstetter et al., 2021; Mann et al., 2023; Koeppe et al., 2023; Graser et al., 2025; Li et al., 2026). In parallel, breeding programs should use RNAi not only as an end-product technology but also as a discovery engine to identify which genes and degrees of suppression create favorable multi-trait states before committing to permanently edited alleles.

In this broader perspective, plant RNAi has moved from being a method to being a design principle. It taught the field that agronomic traits can be improved by rebalancing signaling, not merely by adding or deleting single genes. That lesson remains highly relevant in the age of pangenomes and editing.

## 12 Conclusions

Plant RNA interference has undergone remarkable conceptual development: from unexplained antiviral interference to a mechanistic framework for small-RNA biology, to a practical platform for crop engineering, and now to a delivery-enabled, pangenome-aware component of next-generation breeding. Throughout this history, RNAi repeatedly solved problems that other approaches could not: it overcame gene redundancy, enabled multigene suppression, exposed hidden compensatory networks, and modulated developmental regulators without necessarily destroying function (Agrawal et al., 2003; Lawrence and Pikaard, 2003; Abdurakhmonov et al., 2014, 2016; Miao et al., 2017; Termolino, 2021; Kamburova et al., 2022). For crop improvement, this has profound implications. Some of the most valuable agronomic outcomes do not require permanent elimination of a locus; they require a controlled rebalancing of endogenous pathways.

The future of RNAi-based plant breeding will therefore depend on precision at a level different from that of classic editing. What matters is not only sequence specificity, but also dosage, pathway routing, mobility, tissue context, delivery, and genomic diversity of targets across breeding germplasm. If these dimensions are integrated with pangenomics, phenomics, and genome editing, RNAi should remain a central tool for climate-resilient crop design. Its lasting contribution is the demonstration that a carefully chosen RNA trigger can sometimes improve not just one trait but a coordinated phenotype. For plant biotechnology, that may be the most important lesson of all.

## Glossary

AGO: Argonaute protein: the small-RNA-binding effector that uses guide RNAs to recognize complementary transcripts or chromatin-associated targets
amiRNA: Artificial microRNA: an engineered microRNA precursor designed to produce a defined guide RNA against a chosen plant, pest, pathogen, or viral target
CRISPR: Clustered regularly interspaced short palindromic repeats
in biotechnology: a programmable nuclease or regulatory system used for genome editing, transcriptional regulation, or RNA targeting
DCL: Dicer-like protein: a plant RNase III enzyme that processes double-stranded or structured RNA into small RNAs of characteristic lengths
dsRNA: Double-stranded RNA: a common trigger for RNAi that is processed into small interfering RNAs
HIGS: Host-induced gene silencing: expression of RNAi triggers in a host plant to silence genes in interacting pathogens, pests, nematodes, insects, or parasitic plants
hpRNA: Hairpin RNA: an inverted-repeat RNA construct that folds into dsRNA and triggers RNAi
miRNA: MicroRNA: an endogenous small RNA, usually about 21 nt in plants, that regulates developmental, physiological or stress-responsive transcripts
PTGS: Post-transcriptional gene silencing: RNAi action that primarily degrades or represses translation of target mRNA
RdDM: RNA-directed DNA methylation: a plant pathway in which small RNAs guide DNA methylation and transcriptional silencing at homologous genomic regions
RDR: RNA-dependent RNA polymerase: an enzyme that converts single-stranded RNA into double-stranded RNA, enabling amplification or secondary siRNA production
RNAi: RNA interference: sequence-guided gene silencing triggered by dsRNA or small RNAs and executed through small-RNA effector complexes
siRNA: Small interfering RNA: a small RNA produced from dsRNA that guides sequence-specific silencing
SIGS: Spray-induced gene silencing: exogenous application of dsRNA or small RNAs to induce silencing without stable transformation of the crop
TGS: Transcriptional gene silencing: repression at the DNA or chromatin level, often linked to RdDM in plants
VIGS: Virus-induced gene silencing: transient RNAi triggered by viral vectors used for rapid functional genomics or target validation.
