# Supplementary material for: Plant RNA interference from antiviral silencing to multiplex trait engineering for climate-resilient crops
Source: Front Plant Sci. 2026 Jul 14;17:1871239. doi: 10.3389/fpls.2026.1871239 (PMC13408218; doi:10.3389/fpls.2026.1871239)
Supplement: Supplementary file 1 [file Table1.docx]

**Title:** Plant RNA interference from antiviral silencing to multiplex trait engineering for climate-resilient crops

**Running title:** Plant RNAi for climate-resilient crops

**Authors:** Ibrokhim Y. Abdurakhmonov*

**Supplementary Information**

**SUPPLEMENTARY TABLE S1.** Selected milestone references that shaped the development of plant RNAi concepts and tools.

| Phase | Illustrative milestone references | Why they mattered |
| --- | --- | --- |
| Early interference and co-suppression | Register and Beachy, 1988; Napoli et al., 1990; Rao and Hall, 1991 | Established that viral resistance and homologous transgene/endogene suppression could be sequence-related before RNAi was formally defined. |
| Formal RNAi and small-RNA discovery | Fire et al., 1998; Hamilton and Baulcombe, 1999; Llave et al., 2002; Reinhart et al., 2002 | Connected dsRNA-triggered interference, PTGS, siRNAs, and miRNAs into a small-RNA regulatory framework. |
| RdDM and chromatin-level silencing | Mette et al., 2000; Mathieu and Bender, 2004; Matzke et al., 2009 | Showed that RNA-guided processes could direct methylation and transcriptional gene silencing. |
| Crop and translational RNAi | Kusaba, 2004; Tenllado et al., 2004; Yadav et al., 2011; Xu et al., 2011; Jung et al., 2013 | Moved RNAi into antiviral resistance, metabolic redesign, and lignin/cell-wall engineering. |
| Network and dosage engineering | Schwab et al., 2006; Cui et al., 2010; Chatterjee et al., 2011; Abdurakhmonov et al., 2014; Kamburova et al., 2022 | Showed that partial suppression of developmental or pathway nodes can reveal dosage and compensation. |
| HIGS, SIGS and nanocarriers | Nowara et al., 2010; Koch et al., 2016; Wang et al., 2016; Mitter et al., 2017; Nino-Sanchez et al., 2022 | Expanded RNAi from stable transgenic crops to cross-kingdom silencing, topical dsRNA, and improved formulations. |
| Abiotic stress and climate resilience | Hu et al., 2017; Shanmugam et al., 2021; Lin et al., 2024 | Demonstrated that RNAi can improve drought and salt tolerance through modulation of ROS, stomatal regulation, and hormone signaling in rice, supporting climate-resilience applications. |
| Commercialization and regulation | Darlington et al., 2022; Dietz-Pfeilstetter et al., 2021; OECD, 2020; OECD, 2023; U.S. EPA, 2023a | Established field deployment, risk-assessment questions, and regulatory differentiation among platforms. |

**SUPPLEMENTARY TABLE S2.** Practical design lessons and validation priorities for next-generation RNAi-based plant breeding.

| Design principle | Why it matters | Implication for future breeding | Validation priority |
| --- | --- | --- | --- |
| Define the platform precisely | hpRNA, amiRNA, VIGS, HIGS and SIGS differ in biology, regulation and deployment. | Choose the trigger format based on durability, specificity, mobility, and regulatory context. | Report trigger architecture, delivery route, target sequence, and expected regulatory category. |
| Target pathways, not only single loci | Complex agronomic traits emerge from connected regulatory systems. | Prioritize developmental, defense, wall, or stress nodes with known network reach. | Measure target knockdown plus downstream transcriptomic, biochemical, or physiological effects. |
| Exploit dosage, not maximal silencing. | Intermediate suppression may outperform near-null states. | Screen multiple construct designs, promoters, and expression levels before final selection. | Quantify dose-response and trait trade-offs. |
| Use family- and allele-aware design | Homologs, paralogs and structural variants influence efficacy and off-target risk. | Integrate pangenome and transcriptome resources into target selection. | Perform in silico off-target checks across cultivars and related species. |
| Treat compensation as evidence to test | Compensatory responses may reveal useful network rewiring but can also cause undesirable pleiotropy. | Use RNAi as a discovery engine before fixing alleles by editing or breeding. | Validate molecular compensation across environments and germplasm. |
| Plan deployment early | Laboratory silencing does not guarantee field value. | Assess formulation, field persistence, cost, resistance management, and stewardship. | Include greenhouse, field, multi-season and non-target evaluations as appropriate. |
| Validate durability | hpRNA transgenes can self-silence; sprayed dsRNA can degrade; pest targets can evolve. | Optimize construct architecture and formulation; design resistance-management plans. | Monitor expression, methylation, efficacy and phenotype over multiple seasons. |
